# In Situ Contact Analysis of Polyetheretherketone under Elastohydrodynamic Lubrication

**DOI:** 10.3390/polym14204398

**Published:** 2022-10-18

**Authors:** Stefan Hofmann, Enzo Maier, Thomas Lohner

**Affiliations:** Gear Research Center (FZG), Department of Mechanical Engineering, School of Engineering and Design, Technical University of Munich, Boltzmannstraße 15, 85748 Garching, Germany

**Keywords:** EHL, thermoplastic polymer, in situ, thin-film sensor, twin-disk tribometer

## Abstract

The influence of the complex material behavior of thermoplastic polymers in lubricated contacts is poorly understood. It affects the optimal design of power-transmitting thermoplastic machine elements and the exploitation of its potential, e.g., lightweight design, low-noise operation, and cost-effective manufacturing when injection-molded. This study applies the in situ thin-film sensor technology on a twin-disk tribometer in order to study the elastohydrodynamic lubrication of rolling–sliding contacts with the thermoplastic polymer polyetheretherketone. The results provide insights into the effects and relevance of its thermoplastic material properties. Pressure measurements reveal a typical hydrodynamic profile in combination with a large deformation of the contact zone. The influence of speed and slip ratio is thereby negligible. The temperature rise is low compared to elastohydrodynamically lubricated contacts with steel and is mainly influenced by the slip ratio as well as the load, whereas speed plays a subordinated role. In general, the heat generation is governed by shearing and backflow in the contact inlet zone at low slip ratios and shearing in the contact zone at high slip ratios. No effects attributed to viscoelasticity or loading frequency were observed at the operating conditions considered.

## 1. Introduction

Although polymer materials have been found in almost all machinery for decades, they have only recently become a significant role in power-transmitting machine elements such as spur gears. Technical polymers such as thermoplastics are able to meet the requirements for low noise emission through damping, lightweight design, cost-effective manufacturing and also load-carrying capacity when combined with liquid lubrication. The design of such systems is still a subject of research.

Liquid lubrication reduces friction and wear during contact and removes detrimental heat. Elastohydrodynamically lubricated (EHL) contacts with technical polymers feature approximately one order of magnitude lower contact pressures than steel, which results from the much lower stiffness (approximately two orders of magnitude). As a result, the fluid viscosity increases enough to form a lubricating film between the contact surfaces and remains low enough to reach a very low fluid friction level. The heat generated due to shearing is thus also low and is shown to be in the range of the compression heating according to Vicente et al. [1] as well as Ziegltrum et al. [2]. EHL contacts with technical polymers operate in a transition region between the soft and hard EHL regime, e.g., [3,4,5], and they may reach fluid friction in the range of superlubricity (µ < 0.01) as shown by Reitschuster et al. [6].

The complex and possibly nonlinear dependencies of the material properties of polymers on temperature, humidity, age, loading frequency, and chemical surroundings affect the tribological behavior (Dominghaus [7]) and therefore the design of power-transmitting machine elements. For example, Putignano and Dini [8] show experimentally that the viscoelastic behavior of polymers may cause a pressure maximum at the contact inlet and a film thickness shrinkage at the contact outlet, depending on the operating conditions and the coupling between fluid flow and solid response. This behavior can also result in additional hysteresis friction in the polymer solid, as shown by a strong increase in bulk temperature under pure rolling conditions by Reitschuster et al. [6].

In situ measurements of local quantities in an EHL contact such as the pressure and temperature profiles can offer insights into the complex behavior of polymers during the operation of power-transmitting machine elements. Several in situ measurement principles exist for studying local quantities in EHL contacts [9]. Optical in situ measurement principles are often used. Turchina et al. [10] were among the first to use infrared technology at an optical EHL tribometer to measure contact temperature. The measurement principle has been further developed, especially in the lateral resolution, e.g., by Reddyhoff et al. [11], and it is even possible to measure three-dimensional temperature distribution in the EHL contact (Lu et al. [12]). In addition to temperature measurements using infrared technologies, pressure via Raman microspectroscopy (Jubault et al. [13]) and film thickness using white light interferometry can also be measured. Very few studies have considered technical polymers. Marx, Guegan, and Spikes [14] applied the measurement principle to soft EHL contacts with thermoplastics using polymethylmethacrylate (PMMA) and polyurethane (PU). In addition, Putignano and Dini [8] measured film thickness in an EHL contact with PMMA. All optical in situ measurements require at least one transparent counter body coated with a semi-reflective layer [9].

As a further in situ measurement principle, thin-film sensors can be used to measure temperature, pressure, and film thickness. The pioneering publications are from the early 1960s. Crook et al. [15] were among the first to apply evaporated metallic thin-film sensors to measure the film thickness in EHL contacts based on the capacitive method. Kanel et al. [16] used the pronounced piezoresistive properties of manganin to measure contact pressure using thin-film sensors. Orcutt as well as Chen and Orcutt [17,18] measured the contact temperature with thin-film sensors made of titanium having pronounced thermoresistive properties. Building on these works, numerous authors have applied thin-film sensors for pressure and temperature measurement in hard EHL contacts. The spatial resolution in particular has improved rapidly. Hamilton and Moore [19] resolved the second pressure maximum experimentally. Safa, Anderson, and Leather [20] reduced the active width of a thin-film sensor to 1 µm, thus allowing a high resolution of the second pressure maximum.

Given that the electrical resistance of metallic thin films always depends on pressure and temperature, a mutual interference exists which influences the target value to be measured. Especially with regard to temperature measurements, the influence of contact pressure is not negligible. Ebner et al. [21] report a correction of up to 12 K for platinum thin-film sensors at a maximum contact pressure of 1000 MPa. Numerically calculated pressure profiles were used for pressure correction. In order to align the pressure correction signal, they used the peak concept from Dauber [22] under the assumption of a simultaneous appearance of the second pressure maximum and the maximum contact temperature.

Safa [23] and Baumann [24] developed a twin-layered thin-film sensor made of manganin and titanium. The interference from pressure on the temperature signal of these sensors was able to be reduced by up to 90%, but it is limited due to the complex control of the layer thickness during deposition. Emmrich et al. [25] recently developed thin-film sensors embedded in surface coatings to increase durability and enable measurements under mixed lubrication. Since the surface coatings typically exhibit a lower thermal effusivity than steel, high damping of the measured temperature can occur. Numerical calculations of the temperature distribution in coated EHL contacts from, e.g., Ziegltrum et al. [2] revealed strong temperature gradients within surface coatings. Hofmann et al. [26] applied thin-film sensors to dry lubricated soft-coated rolling-sliding contacts. Beyond rolling–sliding contacts, thin-film sensors have also been investigated for temperature measurement, e.g., in cutting tools [27]. No studies were found, which applied thin-film sensors in EHL contacts with technical polymers.

Technical polymers such as thermoplastics have very different material properties compared to steel. The material behavior can exhibit complex dependencies, and its effect and relevance in EHL contacts is the subject of research. Thin-film sensors are a proven in situ measuring method in hard EHL contacts, but they have yet not been applied to EHL contacts with technical polymers. The objective of this study is to characterize the operating behavior of EHL contacts with polyetheretherketone (PEEK) as a selected thermoplastic polymer by applying thermo- and piezoresistive thin-film sensor technology. The effects and relevance of the thermoplastic material properties of PEEK will be discussed in the context of pressure and temperature measurements.

## 2. Materials and Methods

Thin-film sensor technology is applied in order to measure pressure and temperature in EHL contacts with thermoplastic polymers. The thermo- and piezoresistive concepts only depend on the material properties of the thin-film sensor, thus making the measurand independent of the polymer material properties. PEEK is selected as a promising high-performance polymer for, e.g., power-transmitting gears. The following sections present the twin-disk tribometer, test specimens, and thin-film sensors considered, as well as the operating conditions and experimental procedure.

### 2.1. Twin-Disk Tribometer 

**Figure 1:**
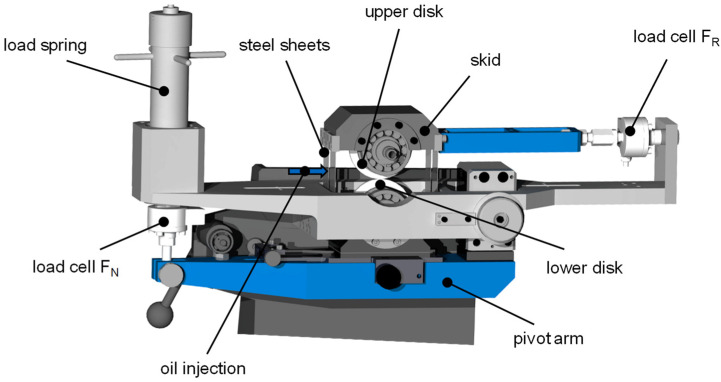
Schematic of the FZG twin-disk tribometer considered based on Ebner et al. Reprinted with permission from Ref. [21]. Copyright 2020 Elsevier.

The experiments are performed on a twin-disk tribometer, which was also used for thin-film sensor measurements in hard EHL contacts by Ebner et al. [21] and in dry lubricated contacts by Hofmann et al. [26]. Figure 1 shows a schematic of the mechanical layout of the twin-disk tribometer considered. The following description and formulations are mainly based on Ebner et al. [21].

The upper and lower disk are pressed-fitted onto shafts, which are individually driven by two three-phase motors. Traction drives mounted between the motor and the driving shafts enable the continuous variation of surface velocities *v*_1_ (upper disk) and *v*_2_ (lower disk). The sum velocity $v_{\sum }$, sliding velocity *v_g_,* slip ratio *s*, and slide-to-roll ratio SRR are defined in Equations (1) to (4).

(1) $$v_{\sum }=v_{1}+v_{2}$$

(2) $$v_{g}=v_{1}-v_{2}$$

(3) $$s=\frac{\left(v_{1}-v_{2}\right)}{v_{1}}\;\cdot 100\%\;\mathrm{with}\;v_{1}>v_{2}$$

(4) $$\mathrm{SRR}=\frac{2\cdot \left(v_{1}-v_{2}\right)}{\left(v_{1}+v_{2}\right)}=\frac{2\cdot v_{g}}{v_{\sum }}$$

The normal force *F_N_* during disk contact is continuously applied by a load spring via a pivot arm where the lower disk is mounted. The upper disk is mounted in a skid, which is attached to the frame by thin steel sheets. The skid is supported laterally by a load cell so that the friction force *F_R_* in the disk contact for slip ratio *s* ≠ 0% can be measured as a reaction force with hardly any skid displacement. The upper disk *v*_1_ is thereby faster than the lower disk *v*_2_. The normal force *F_N_*, friction force *F_R_*, surface velocities *v*_1_ and *v*_2_, and the bulk temperature $\vartheta_{M}$ of the test disk 5 mm below the surface are measured. An injection lubrication unit is used for conditioning the oil temperature and oil volume. The lubricant is injected directly into the contact inlet with the oil temperature $\vartheta_{oil}$ at a volume flow of $\dot{v}$ = 1.5 L/min. In order to provide an evenly distributed load during line contact of the disks, a contact print on pressure indicating sensor film (Fujifilm Prescale^®^) is carefully evaluated before each test, and any misalignment is corrected mechanically. 

### 2.2. Test Specimens and Material Data 

**Figure 2:**
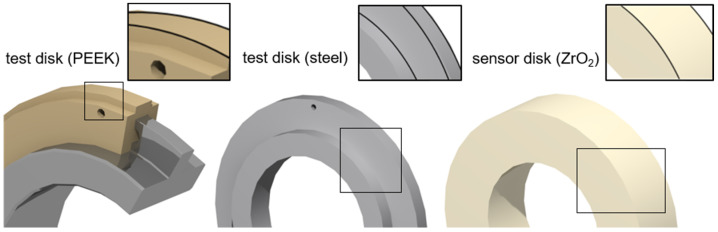
Schematic representation of the considered test disks (width of 5 mm) and sensor disk (width of 20 mm).

An electrically insulating ceramic sensor disk made of zirconium dioxide (ZrO_2_) is used as the upper disk in all of the experiments. The thin-film sensors described in Section 2.3 are applied to its surface. The lower test disk is made of PEEK or case-hardened steel 16MnCr5 (AISI5115) for reference measurements. The material pairing ZrO_2_/PEEK refers to thermoplastic EHL contact, and the material pairing ZrO_2_/steel refers to steel EHL contact. The PEEK test disk is injection-molded around a perforated steel inlay, as described by Reitschuster et al. [6]. PEEK was chosen as a promising material for power-transmitting machine elements because it exhibits stable material properties over a wide range of temperatures [7]. Figure 2 shows a schematic representation of each test and sensor disk. The disks have a diameter of 80 mm and a width of 5 mm or 20 mm, respectively.

All surfaces are mechanically polished to a mean arithmetic surface roughness Ra ≤ 0.03 µm. Roughness measurements were performed in the axial direction by the profile method according to DIN EN ISO 13565-1 to 13565-3, with a measured length of *L_t_* = 4.0 mm and a cut-off wavelength of $\lambda_{c}$ = 0.08 mm. Table 1 clarifies the mechanical and thermophysical properties of the test and sensor disks considered.

### 2.3. Thin-Film Sensors

**Figure 3:**
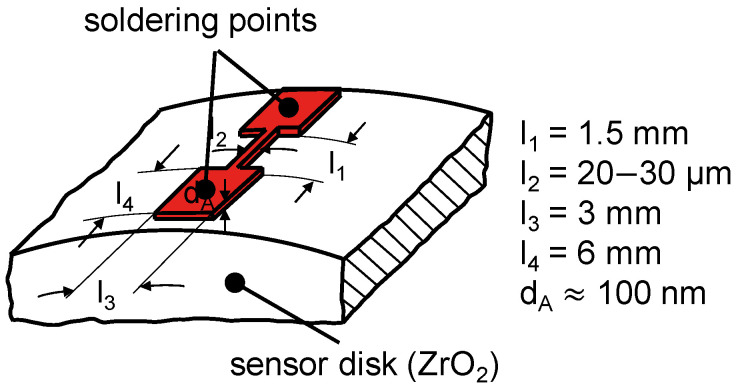
Layout of the applied thin-film sensors.

Thin-film sensors are applied on ceramic sensor disks by means of process photolithography for masking and ion beam sputtering for the deposition process. The manufacturing process is described in detail by Ebner et al. [21]. Figure 3 shows the typical layout of the thin-film sensors applied at a total layer thickness *d_A_* of between 100 and 150 nm in order to minimize the sensor influence on the contact quantities [31].

Given its high sensitivity to temperature and low sensitivity to pressure, platinum is used for the temperature measurements. To increase the adhesion between the thin-film sensor and the ceramic sensor disk, a titanium layer of approximately 40 nm was applied at NMI Reutlingen, Reutlingen, Germany. The width *l_2_* of the active part of the thin-film sensor is approximately 20 µm. The ohmic resistance of a platinum thin-film sensor in the manufacturing state is in the range of *R* = 120 Ω. Given its high sensitivity to pressure and weak sensitivity to temperature, chromium is used for the pressure measurements. The sensor was manufactured in cooperation with NMI Reutlingen, Reutlingen, Germany. The width *l_2_* of the active part of the thin-film sensor is approximately 30 µm. The ohmic resistance in the manufacturing state is in the range of *R* = 1000 Ω, which enables a higher supply voltage of the measurement chain [32] and thus improves the signal-to-noise ratio. Note that the electrical properties of thin chromium films depend on the geometry and the deposition process. 

The correlation between the resistance change $\frac{\Delta R}{R_{0}}$ of thin-film sensors due to temperature ΔT and pressure Δp can be expressed by

(5) $$\frac{\Delta R}{R_{0}}=\alpha_{T}\cdot \Delta T+\alpha_{p}\cdot \Delta p$$

with $\alpha_{T}$ as the temperature coefficient and $\alpha_{P}$ as the pressure coefficient. The ratio *k* between the temperature- and pressure-dependent resistance change can be used to evaluate the suitability of a sensor material for pressure or temperature measurement. Note that this suitability depends on both the material properties and the absolute increases in temperature and pressure. The latter increase is particularly influenced by the contact type as well as the operating conditions conducted.

(6) $$k=\frac{\left|\alpha_{T}\cdot \Delta T\right|}{\left|\alpha_{p}\cdot \Delta p\right|}$$

A ratio of *k* ≫ 1 is suitable for temperature measurements, and *k* ≪ 1 is suitable for pressure measurements. The temperature coefficients for the thin-film sensors were derived by way of calibration in a tempered oil bath with the lubricant considered (cf. Section 2.4). In addition to the ohmic resistance, the output voltage of the amplifier was also tracked for the platinum sensor to exclude thermoelectric effects between the titanium adhesion layer. The pressure coefficient for the platinum sensor was derived by nearly steady roll-overs at the twin-disk tribometer as reported, e.g., in Emmrich et al. [25]. The pressure coefficient of the chromium sensor was derived based on the equilibrium of integrated contact pressure over the sensor distance and external load *F_N_* (see, e.g., Hamilton and Moore [19]). Pressure profiles at pure rolling were chosen in order to minimize the influence of temperature on resistance change. Note that this method is only suitable for pressure sensors given the low interference of temperature on sensor resistance. The resulting temperature and pressure coefficients of the thin-film sensors are summarized in Table 2.

**Figure 4:**
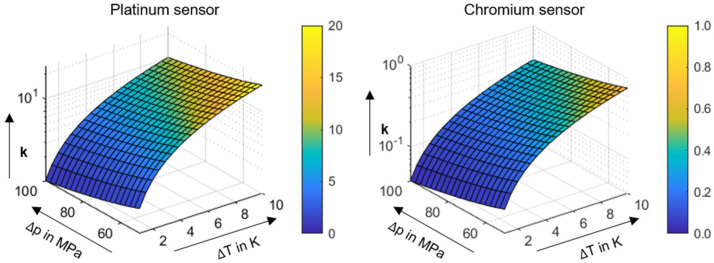
Ratio *k* of temperature and pressure-dependent resistance change for the platinum (**left**) and chromium (**right**) thin-film sensor.

Based on the temperature and pressure coefficients, Figure 4 shows the ratio *k* as a function of the absolute pressure Δ*p* and temperature change Δ*T*. In this case, Δ*p* and Δ*T* are adapted to the expected changes in the thermoplastic EHL contact with ZrO_2_/PEEK.

Regarding the investigated material pairing and operating conditions (cf. Section 2.4), the platinum thin-film sensor for temperature and chromium thin-film sensor for pressure measurement demonstrate satisfactory suitability in terms of the measured temperatures and pressures (cf. Section 3). The platinum thin-film sensor *k* exhibits its minimum of *k* = 2.97 at w = 150 N/mm, $v_{\sum }$ = 8 m/s and pure rolling (*s* = 0%), and the chromium thin-film senor has its maximum of *k* = 0.39 at *w* = 100 N/mm, $v_{\sum }$ = 12 m/s and *s* = 50%. This result supports the application of thin-film sensor technology on thermoplastic contacts. Note that the temperature and pressure scales are nearly identical within soft and hard EHL contacts (cf. Section 3.3), thus resulting in a comparable *k* ratio.

### 2.4. Operating Conditions and Lubricant 

Table 3 illustrates the operating conditions considered, which are derived from the kinematic conditions typical of plastic gears, e.g., Illenberger, Tobie, and Stahl [33]. The slip ratios *s* considered corresponds to an SRR of between 0 and 0.66.

Mineral oil (ISO VG 100) with 4% sulfur–phosphorus-based extreme-pressure (EP) additive Anglamol A99 is used as lubricant, the main properties of which are described in Table 4 [34].

The lubrication regime was estimated based on the relative film thickness $\lambda_{rel}$ according to Niemann et al. [35] and at the minimum film thickness h_m_ according to Myers et al. [3] for the PEEK test disk and Dowson and Higginson [36] for the steel test disk. Fluid film lubrication with $\lambda_{rel}$ >> 2 is observed in all of the operating conditions considered. To classify EHL contacts, Johnson [37] introduced a viscosity (g_1_) and elasticity parameter (g_3_), depending on the material and lubricant properties as well as the operating conditions. Therefore, given g_1_ = {35.2–109.6} and g_3_ = {10.3–28.1}, the thermoplastic EHL contact ZrO_2_/PEEK considered operates in the transition regime. In the reference steel EHL contact ZrO_2_/steel, the viscosity parameter g_1_ range is g_1_ = {44.6–80.9}, and the elasticity parameter range is g_3_ = {2.3–3.5}. Hence, it operates in the rigid piezoviscous regime.

### 2.5. Experimental Procedure and Evaluation

The measurement chain is calibrated at room temperature before each test sequence. The kinematic conditions are initially adjusted for each operating point at the twin-disk tribometer, with the sensor and test disks separated from each other. When the bulk temperature $\vartheta_{M}$ of the test disk is basically quasi-stationary, the normal load *F_N_* is applied, and eight roll-overs are tracked at a sampling rate of 2 MS/s. This procedure is repeated once, resulting in 16 recorded roll-overs for every operating point. As a result, all of the pressure and temperature profiles described in this study represent a mean value of 16 signals. Each test sequence is started at $v_{\sum }$ = 8 m/s and pure rolling (*s* = 0%). After increasing the slip ratio up to *s* = 50%, the procedure is repeated for $v_{\sum }$ = 12 m/s and 16 m/s. The tribometer is cooled down to room temperature, and the measurement chain is calibrated again when conducting a different line load w.

Every single signal is filtered using a low-pass filter, whereby the cut-off frequency is adapted to the sensor width *l*_2_ based on the formulations of Marko [38]. To better visualize the various speeds and slip ratios, the time is converted to the sensor disk distance *x*. Regarding the contact temperature measurements, the temperature rise is in reference to the minimum temperature measured before the contact zone (cf. Section 3.2.1). As a result, the heat generation due to shearing of the lubricant in the inlet and contact zone, as well as due to compression, are able to be compared and related to the operating conditions under consideration. The surface temperature $\vartheta_{S}$ of the sensor disk is able to be obtained during conditioning. Note that this is only possible in operating conditions under full film lubrication and no sensor wear. The latter would result in resistance change and hence pseudo temperature rise.

## 3. Results

The description of experimental results is divided into contact pressure (Section 3.1) and contact temperature (Section 3.2). The focus is put on contact temperature, including the general characteristics of the thermoplastic EHL contact, as well as the influences of slip ratio, load, and speed. Section 3.3 presents the results of a reference steel EHL contact at the same line load.

### 3.1. Contact Pressure

**Figure 5:**
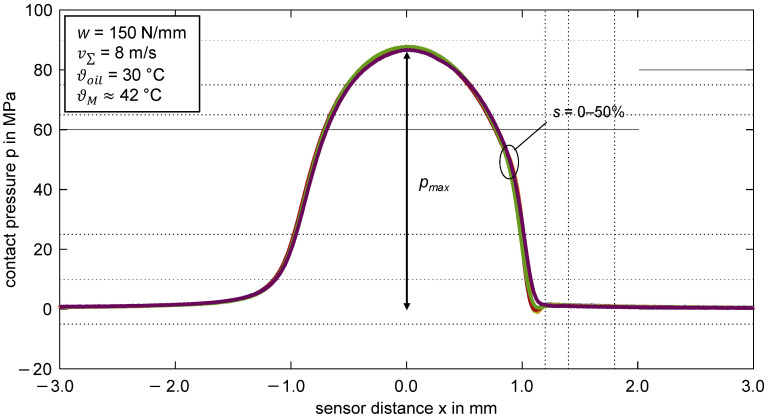
Measured contact pressure for the thermoplastic EHL contact ZrO_2_/PEEK (line load *w* = 150 N/mm, sum velocity $v_{\sum }$ = 8 m/s, slip ratios *s* = {0; 5; 10; 20; 30; 40; 50}%, oil temperature $\vartheta_{oil}$ = 30 °C, MIN100).

Figure 5 shows the pressure profiles measured over the sensor distance *x* for the thermoplastic EHL contact ZrO_2_/PEEK at a line load of *w* = 150 N/mm, a sum velocity of v_Σ_ = 8 m/s, and slip ratios of *s* = {0; 5; 10; 20; 30; 40; 50}%. The maximum contact pressure *p_max_* is aligned with the sensor distance *x* = 0 mm. The thin-film sensor made of chromium was used.

The pressure profiles show a hydrodynamic pressure build-up starting at a sensor distance of *x* ≈ −2.5 mm in the contact inlet zone. The pressure profiles in the contact zone resemble the Hertzian pressure profile at a maximum pressure of *p_max_* ≈ 87 MPa. A second pressure maximum such as that of hard EHL contacts [22,32] is not visible. The absence of a second pressure peak is typically for soft EHL contacts [2] and not caused by insufficient spatial resolution of the sensor. The width of the pressurized zone of around ≈ 2.2 mm refers to the large deformation of the PEEK surface. In comparison of the slip ratios, there is no remarkable difference in the pressure distribution at the operating points considered. Note that the bulk temperature of the PEEK disk is higher than the oil injection temperature, which can be attributed to the mechanical setup of the twin-disk tribometer (cf. Section 3.2.1). For the other investigated operating points, the schematic profile of the pressure distribution is similar to that shown in Figure 5. The influence of line load *w* and sum velocity $v_{\sum }$ is discussed in Section 4.1.

### 3.2. Contact Temperature

The contact temperature characteristics of the thermoplastic EHL contact ZrO_2_/PEEK as well as the influence of slip ratio, load, and speed are described in the following. The thin-film sensor made of platinum was used.

#### 3.2.1. Characteristics

**Figure 6:**
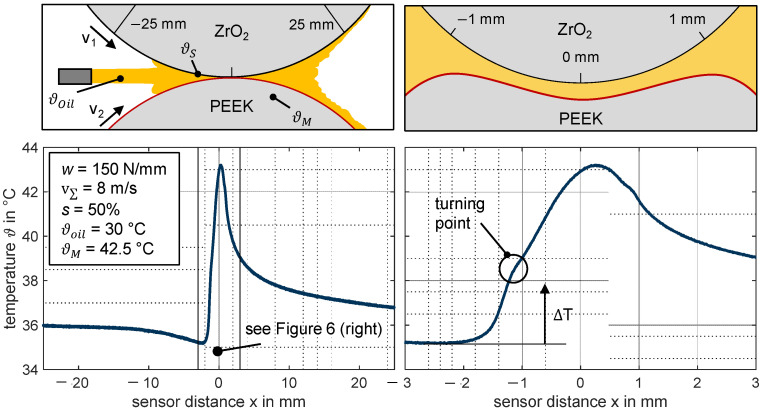
Exemplarily measured temperature profile with an illustration of the trajectory on the sensor disk considered with magnification of the contact area for the thermoplastic EHL contact ZrO_2_/PEEK without pressure correction (line load *w* = 150 N/mm, sum velocity $v_{\sum }$ = 8 m/s, slip ratio *s* = 50%, oil temperature $\vartheta_{oil}$ = 30 °C, MIN100).

Figure 6 shows, by way of example, a measured contact temperature profile along with a schematic representation of the considered trajectory of the sensor distance for the thermoplastic EHL contact ZrO_2_/PEEK. Note that the measured contact temperature refers to the temperature near the ceramic sensor disk. Given the differing thermal effusivities of ZrO_2_ and PEEK, it can be assumed that the temperature near the PEEK test disk is higher. Numerical calculations by Ebner et al. [21] and Ziegltrum et al. [2] reveal the strong temperature gradients in the gap height direction. The deformation illustrated in Figure 6 is derived from numerical calculations by Ziegltrum et al. [2]. The temperature profile refers to the contact temperature *ϑ* over the sensor distance *x* at a line load of *w* = 150 N/mm, sum velocity of $v_{\sum }$ = 8 m/s, and a slip ratio of *s* = 50% without pressure correction. The profile in Figure 6 (left) is shown over a tracked large sensor distance *x* from −25 to +25 mm. A detailed part with a sensor distance x from −3 to +3 mm is shown in Figure 6 (right). The contact temperature ϑ is determined according to Equation (7) as the sum of measured temperature rise Δ*T* and the surface temperature $\vartheta_{S}$ of the sensor disk. The latter is measured during tribometer conditioning without disk contact (cf. Section 2.5).

(7) $$\vartheta =\vartheta_{S}+\Delta T$$

The bulk temperature $\vartheta_{M}$ of the test disk and surface temperature $\vartheta_{S}$ of the sensor disk being higher than the oil injection temperature $\vartheta_{oil}$ is mainly due to high no-load losses of the roller bearings at the twin-disk tribometer. In this case, the lower thermal effusivity of PEEK results in a $\vartheta_{M}$ even higher than the $\vartheta_{S}$ of the ceramic sensor disk. The higher the rotational speed of the shafts, the higher the bulk and surface temperature becomes.

**Figure 7:**
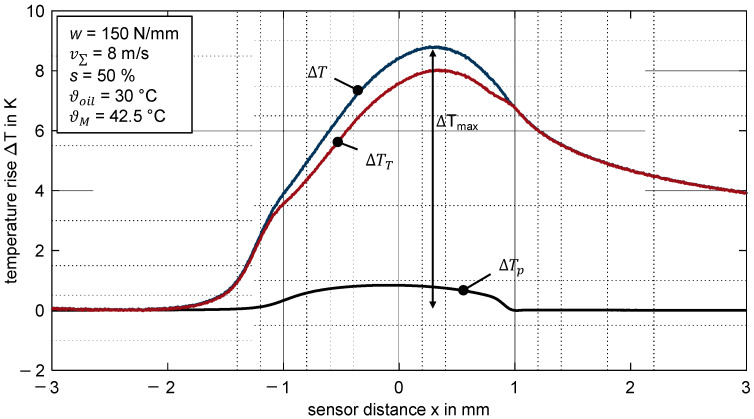
Measured temperature rise $\Delta T_{T}$, pressure-induced correction temperature $\Delta T_{p}$ and pressure-corrected temperature rise ΔT for the thermoplastic EHL contact ZrO_2_/PEEK (line load *w* = 150 N/mm, sum velocity $v_{\sum }$ = 8 m/s, slip ratio *s* = 0%, oil temperature $\vartheta_{oil}$ = 30 °C, MIN100).

**Figure 8:**
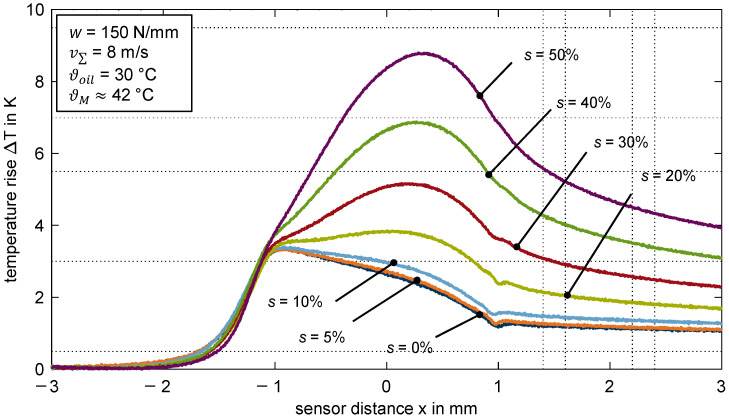
Temperature rise ΔT for different slip ratio *s* = {0; 5; 10; 20; 30; 40; 50}% for the thermoplastic EHL contact ZrO_2_/PEEK (line load *w* = 150 N/mm, sum velocity $v_{\sum }$ = 8 m/s, oil temperature $\vartheta_{oil}$ = 30 °C, MIN100).

The profile, shown by way of example, can be divided into characteristic areas. When the thin-film sensor approaches the contact (*x* ≈ −15…−3 mm), the temperature is decreasing. This result correlates with the higher stationary surface temperature ($\vartheta_{S}>\vartheta_{Oil}$) of the sensor disk compared to the oil injection temperature at the operating conditions investigated. Therefore, the injected oil cools the surface of the sensor disk, which is an effect that becomes pronounced close to the contact inlet zone. After reaching a minimum (*x* ≈ −3 mm), the temperature rises due to both shearing in the inlet and backflow and subsequently due to shearing and compression of the lubricant in the contact zone. The transition from the inlet to contact zone is interpreted as a turning point in the measured temperature signal (cf. Figure 6, right). The maximum temperature rise Δ*T_max_* (cf. Figure 7) occurs in the contact zone. Note that a decrease in temperature in the contact zone is observed at lower slip ratios (cf. Figure 8), so the maximum temperature rise can occur at the transition point from the inlet to the contact zone. After the thin-film sensor left the contact (*x* ≈ 1 mm), the contact temperature exhibits a typical exponential fading.

#### 3.2.2. Pressure Correction

The ohmic resistance of platinum exhibits a high level of temperature sensitivity (cf. Section 2.3). Nevertheless, pressure cannot be neglected because the pressure rise is relevant. In order to correct the influence of pressure on the temperature profile measured, a correction signal $\Delta T_{p}$ is derived directly from Equation (5) and added to the non-corrected temperature profile $\Delta T_{T}$ according to Equation (9).

(8) $$\Delta T_{p}=\frac{\alpha_{p}}{\alpha_{T}}\cdot \Delta p$$

(9) $$\Delta T=\Delta T_{T}+\Delta T_{p}\;$$

The measured pressure profiles described in Section 3.1 are used for pressure correction of the measured contact temperatures. Regarding the positioning of the pressure correction signal with the temperature signal, Dauber [22] suggests four different approaches for hard EHL contacts. Given that no second pressure maximum is observed at the end of the contact zone for the considered thermoplastic EHL contact (cf. Section 3.1), these concepts cannot be applied. Therefore, an alternative approach is used. After the thin-film sensor has passed through the EHL contact zone, the temperature profile shows a very characteristic fading. At the beginning of this exponential profile, the pressure is defined as zero so that the pressure profile is aligned to this point.

By way of example, Figure 7 shows, for a line load of *w* = 150 N/mm, a sum velocity of $v_{\sum }$ = 8 m/s and a slip ratio of *s* = 50%, the pressure-induced correction temperature rise $\Delta T_{p}$ as well as the uncorrected $\Delta T_{T}$ and corrected ΔT contact temperature profile. Note that Figure 7 refers to the measurement signal shown in Figure 6, from *x* = −3 mm to *x* = 3 mm.

Figure 7 shows that the transition from hydrodynamic pressure build-up to the nearly elliptical shape (cf. Figure 5) fits well with the turning point of the temperature profile (*x* ≈ −1 mm). Furthermore, the pressure correction leads to a steady temperature profile in the outlet zone, and the width of the pressure profile agrees with the width of the temperature profile. Details about the thermal effects within the inlet and contact zone are discussed in Section 4.2. All further temperature profiles ΔT shown in Section 3 and Section 4 are pressure corrected by using the measured pressure signals under pure rolling conditions at the sum velocity considered (cf. Section 3.1).

#### 3.2.3. Influence of Slip Ratio

Figure 8 shows the measured temperature rise over the sensor distance x for the thermoplastic EHL contact ZrO_2_/PEEK for various slip ratios *s*. The line load and sum velocity is constant at *w* = 150 N/mm and $v_{\sum }$ = 8 m/s.

There is no remarkable difference in the temperature profiles for pure rolling and low slip ratios of *s* = {0; 5; 10}%. The maximum temperature rise $\Delta T_{max}$ is around 3.4 K and occurs at the inlet zone of the contact. At slip ratios of *s* ≥ 20%, the maximum temperature rise occurs in the contact zone, increases steadily, and reaches a maximum of $\Delta T_{max}$ = 8.8 K at *s* = 50%. Note that the temperature rises in the inlet and contact zone have different underlying mechanisms. The temperature rise in the inlet zone does not depend on the slip ratio, whereas the temperature rise in the contact zone depends on the slip ratio and thus on the shearing of the lubricant. See Section 4.2 for further details.

#### 3.2.4. Influence of Load

**Figure 9:**
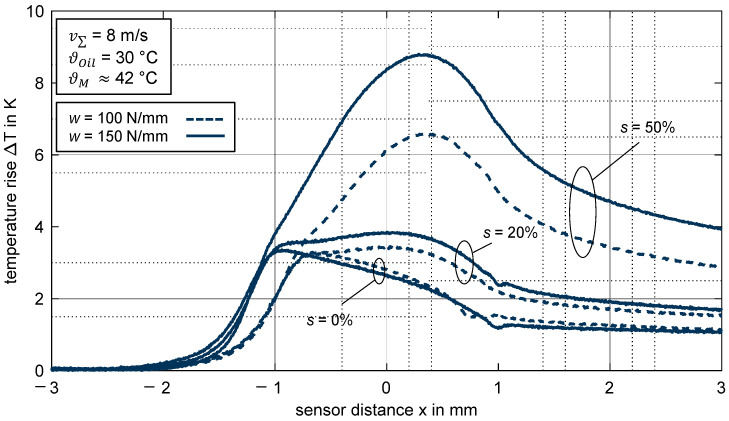
Temperature rise ΔT for different line loads *w* = {100; 150} N/mm for the thermoplastic EHL contact ZrO_2_/PEEK (slip ratio *s* = {0; 20; 50}%, sum velocity $v_{\sum }$ = 8 m/s, oil temperature $\vartheta_{oil}$ = 30 °C, MIN100).

Figure 9 shows the measured temperature rise over the sensor distance x for the thermoplastic EHL contact ZrO_2_/PEEK in comparison to the line loads *w* = {100; 150} N/mm. The sum velocity and the slip ratios are constant at $v_{\sum }$ = 8 m/s and *s* = {0; 20; 50}%. Since the sensor distance is presented with units, the contact widths are different for *w* = 100 N/mm and w = 150 N/mm.

For pure rolling with *s* = 0%, the maximum temperature rise is nearly identical for both considered line loads w. In the respective contact inlet zones, the temperature profiles match and even in the contact zone, the profiles are similar considering different contact widths. For the slip ratio of *s* = 20%, the temperature rise in the inlet zone is also nearly identical and reaches a slightly higher value for the line load of *w* = 150 N/mm. In the contact zone, the temperature rise for the higher line load *w* is moderately higher, whereby the maximum absolute difference is around 0.5 K. Nevertheless, the qualitative profiles show the same trend, as $\Delta T_{max}$ is only slightly higher in the middle of the contact compared to the inlet zone. Remarkable heat generation in the contact zone can be only observed for the high slip ratio of *s* = 50%, whereby the temperature rise is more pronounced for the higher line load of *w* = 150 N/mm with $\Delta T_{max}$ = 8.8 K compared to the lower line load *w* = 100 N/mm with $\Delta T_{max}$ = 6.6 K.

#### 3.2.5. Influence of Speed

**Figure 10:**
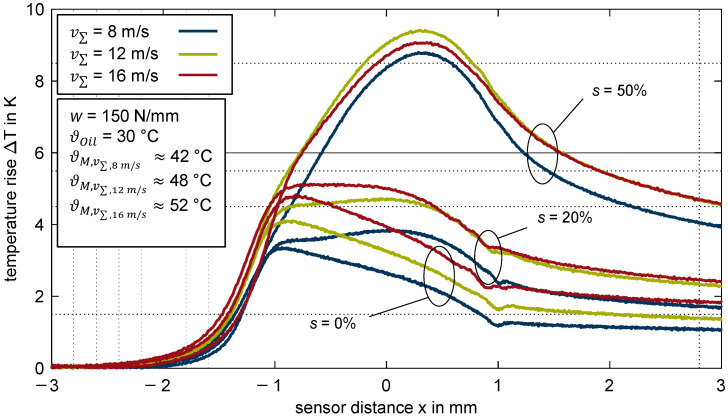
Temperature rise ΔT for different sum velocities $v_{\sum }$ = {8; 12; 16} m/s for the thermoplastic EHL contact ZrO_2_/PEEK (slip ratio *s* = {0; 20; 50}%, line load *w* = 150 N/mm, oil temperature $\vartheta_{oil}$ = 30 °C, MIN100).

Figure 10 shows the measured temperature rise over the sensor distance *x* for the thermoplastic EHL contact ZrO_2_/PEEK in comparison of the sum velocities *v*_Σ_ = {8; 12; 16} m/s. The line load and slip ratios are constant at w = 150 N/mm and *s* = {0; 20; 50}%.

For pure rolling with *s* = 0%, $\Delta T_{max}$ in the contact inlet zone increases from 3.4 K for *v_Σ_* = 8 m/s to 4.8 K for *v_Σ_* = 16 m/s. After reaching the maximum temperature rise, the temperature decreases and the profiles follow a very similar trend. At *s* = 20%, the maximum temperature rise increases with the sum velocity, whereby the temperature rise in the inlet and contact zone is on a similar level. At *s* = 50%, the maximum temperature rise occurs in the contact zone and is about 8.8 K for *v_Σ_* = 8 m/s, 9.4 K for *v_Σ_* = 12 m/s, and 9.1 K for *v_Σ_* = 16 m/s. As a result, the maximum temperature rise does not exhibit a high relevant level of dependency on the sum velocity at high slip ratios. Note that the temperature rise in the inlet zone was barely affected by the slip ratio (see Section 3.2.3).

### 3.3. Pressure and Temperature Profiles of Hard EHL Contacts

**Figure 11:**
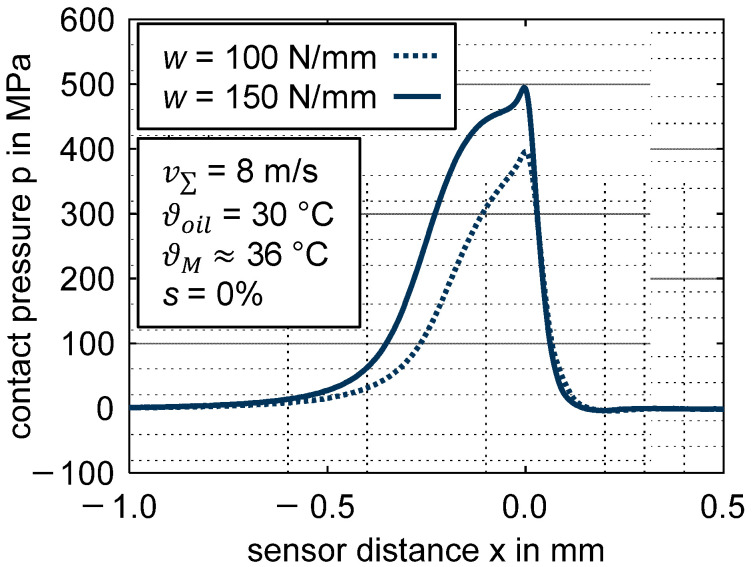
Contact pressure p for different line loads *w* = {100; 150} N/mm for the steel EHL contact ZrO_2_/steel (slip ratio *s* = 0%, sum velocity $v_{\sum }$ = 8 m/s, oil temperature $\vartheta_{oil}$ = 30 °C, MIN100).

Reference measurements using the ZrO_2_/steel disk pairing are conducted in order to compare the thermoplastic EHL contact with typical hard EHL contacts. The same line load *w* is considered. Figure 11 shows the measured contact pressures for $v_{\sum }$ = 8 m/s and *s* = 0% in comparison with the line loads *w* = {100; 150} N/mm. The pressure profiles demonstrate a typical hydrodynamic pressure build-up, which is followed by a rapid pressure increase to the maximum contact pressure *p_max_*. After reaching p_max_, the pressure drops to zero. The pressure profiles agree well with existing studies on hard EHL contacts using thin-film sensor technology [22,32,39] or numerically calculated pressure profiles [2].

**Figure 12:**
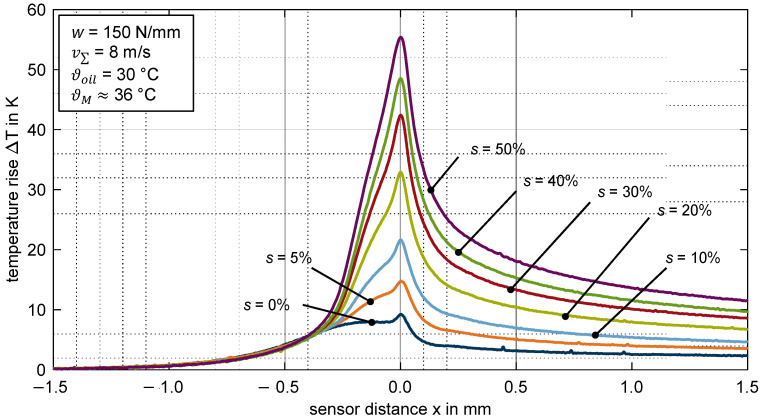
Temperature rise ΔT for different slip ratios where *s* = {0; 5; 10; 20; 30; 40; 50}% for the steel EHL contact ZrO_2_/steel (line load *w* = 150 N/mm, sum velocity $v_{\sum }$ = 8 m/s, oil temperature $\vartheta_{oil}$ = 30 °C, MIN100).

Temperature measurements are performed for *w* = 150 N/mm, $v_{\sum }$ = 8 m/s, and *s* = {0; 5; 10; 20; 30; 40; 50}%. Figure 12 shows the corresponding temperature profiles with a pressure correction based on the measured pressure profile for *w* = 150 N/mm shown in Figure 11. The peak concept from Dauber [22] was used for signal alignment. The temperature profiles show an increase in the inlet zone during the hydrodynamic pressure build-up of approximately ≈5 K, which is mainly due to compression of the oil. For pure rolling (*s* = 0%), the temperature shows almost no increase within the contact zone. Under rolling–sliding conditions, the temperature increases with an increasing slip ratio, which is due to lubricant shearing. The maximum temperature rise is reached for *s* = 50% with 55 K, which is much higher than that of the thermoplastic EHL contact.

## 4. Discussion

All of the results obtained from in situ pressure and temperature measurements in the thermoplastic EHL contact are discussed in the following. A comparison to a reference steel EHL contact completes the discussion.

### 4.1. Analysis of Pressure Trends

**Figure 13:**
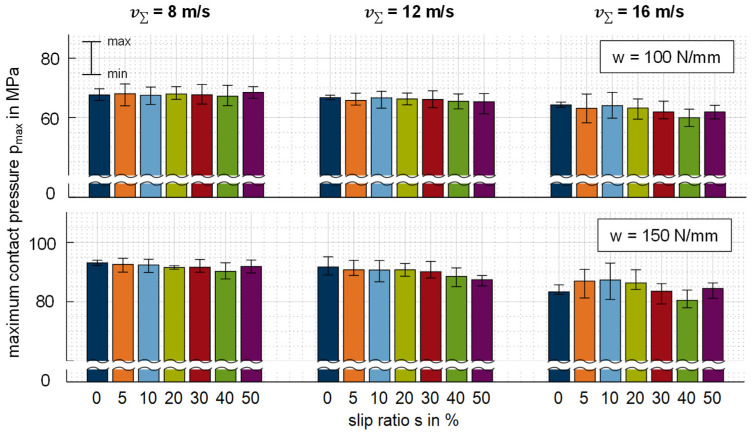
Evaluated maximum contact pressure *p_max_* for the thermoplastic EHL contact ZrO_2_/PEEK in comparison with various line loads w, sum velocities v_Σ_ and slip ratios s.

Figure 13 shows a comparison of the maximum contact pressures *p_max_* evaluated based on the measured pressure profiles for the thermoplastic EHL contact (cf. Figure 5). The signal scattering refers to the minimum and maximum value obtained based on 16 single measurements.

Regarding the sum velocity of $v_{\sum }$ = 8 m/s, *p_max_* is nearly constant over the slip ratio s for both line loads *w* considered. This trend is also observed for $v_{\sum }$ = 12 m/s, whereby *p_max_* slightly decreases at a line load of *w* = 150 N/mm and high slip ratios. For $v_{\sum }$ = 16 m/s, *p_max_* is slightly lower at both line loads, and the scattering is higher than at $v_{\sum }$ = {8; 12} m/s, which may have been due to dynamic effects at the twin-disk tribometer considered. In general, the measured contact pressures in combination with the large deformation (cf. Section 3.1) are typical for thermoplastic EHL contacts [5] related to the transition regime (cf. Section 2.4) according to Johnson [37]. Moreover, the pressure profile findings (cf. Section 3.1) agree with numerical results for thermoplastic EHL with PEEK by, for example, Ziegltrum et al. [2] at the operating conditions considered. In contrast to viscoelastic effects observed with, for example, PMMA [8], the measured pressure profiles do not reveal significant viscoelastic effect or loading frequency influence.

Note that the chromium thin-film sensor shows a weak temperature dependency on sensor resistance (cf. Section 2.3). The maximum error when disregarding the thermoresistive influence is between 0.15 MPa (*s* = 0%) and 0.3 MPa for the highest measured temperature rise at *s* = 50%.

### 4.2. Analysis of Temperature Trends

**Figure 14:**
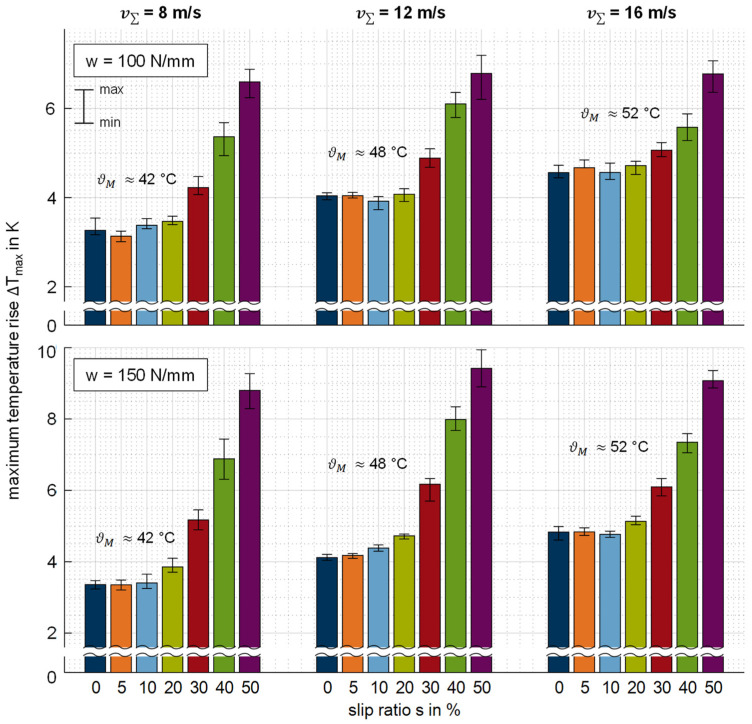
Evaluated maximum temperature rise $\Delta T_{max}$ for the thermoplastic EHL contact ZrO_2_/PEEK in comparison with various line loads *w*, sum velocities v_Σ_, and slip ratios *s*.

In order to evaluate the thermal behavior of the thermoplastic EHL contact, the maximum temperature rise $\Delta T_{max}$ for each operating condition is derived from the measured temperature profiles. Figure 14 shows the comparison of the investigated speed and load variation at the conducted slip ratios. The signal scattering shown ranges from the minimum to the maximum value obtained for 16 single measurements.

The maximum temperature rise $\Delta T_{max}$ is very similar for slip ratios *s* = {0; 5; 10}% at all of the loads and speeds considered. This increase occurs at the inlet zone (cf. Section 3.2) and increases with sum velocity $v_{\sum }$. For slip ratios *s* > 10%, $\Delta T_{max}$ increases with the slip ratio and occurs in the contact zone (cf. Section 3.2). Ziegltrum et al. [2] report a similar magnitude of heat sources due to compression and shearing in thermoplastic EHL contacts. Hence, at a low slip ratio, the heat source due to shearing and backflow at the contact inlet is dominant, whereas at higher slip ratios, the heat source due to shearing at the contact zone is dominant. The scattering is very low, especially at the slip ratios up to 10%, and it increases slightly at the higher slip ratios.

The maximum temperature rise at high slip ratios does not increase significantly with increasing sum velocity. This indicates that the thermal behavior of the thermoplastic EHL contact does not correspond to the sliding velocity. At both line loads w, Δ*T_max_* is reached at $v_{\sum }$ = 12 m/s and *s* = 50%. In this context, it should be noted that the thermal boundary conditions are different for the sum velocities considered because the bearing power losses in the shafts increases for higher sum velocities. This increases the bulk temperature $\vartheta_{M}$ of the PEEK test disk from ≈42 °C at $v_{\sum }$ = 8 m/s to ≈52 °C at $v_{\sum }$ = 16 m/s. Comparable behavior takes place with respect to the surface temperature of the sensor disk. As a result, the mean temperature in the EHL contact increases and leads to lower effective viscosity and thus friction. As expected, the variation of load shows higher temperature rises at the higher line load due to friction power.

All in all, the maximum temperature rise is $\Delta T_{max}$ < 10 K for all operating conditions, despite the low thermal effusivity (see Table 1). Highly loaded hard EHL contacts, e.g., investigated from [21,22,23,39], feature significantly higher contact temperatures at the same line load (cf. Section 4.3). This clearly shows that the friction in thermoplastic EHL contacts is low and thus results in limited heat generation. Investigations by Reitschuster et al. [6] on a twin-disk tribometer under comparable operating conditions indicate coefficients of friction of µ < 0.01. In contrast to highly loaded steel contacts, the material properties of thermoplastics can show a pronounced temperature dependency such that the low temperature rise during contact is favorable to operation. At the operating conditions considered, the bulk temperature $\vartheta_{M}$ of the PEEK test disk, in combination with the low temperature rise ΔT during contact, does not affect the stiffness of the material [7].

### 4.3. Comparison with Steel EHL Contact

**Figure 15:**
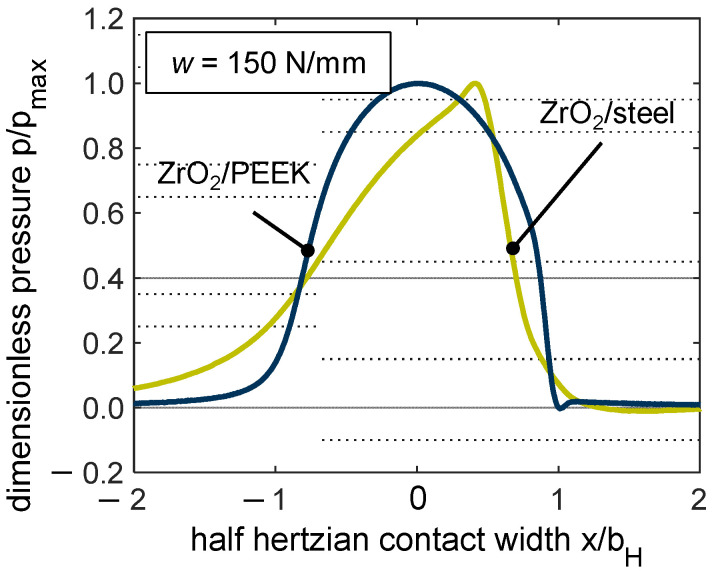
Comparison of the pressure profile for the thermoplastic EHL contact PEEK/ZrO_2_ (blue) and steel EHL contact steel/ZrO_2_ (green) for line load *w* = 150 N/mm, $v_{\sum }$ = 8 m/s and *s* = 0%.

**Figure 16:**
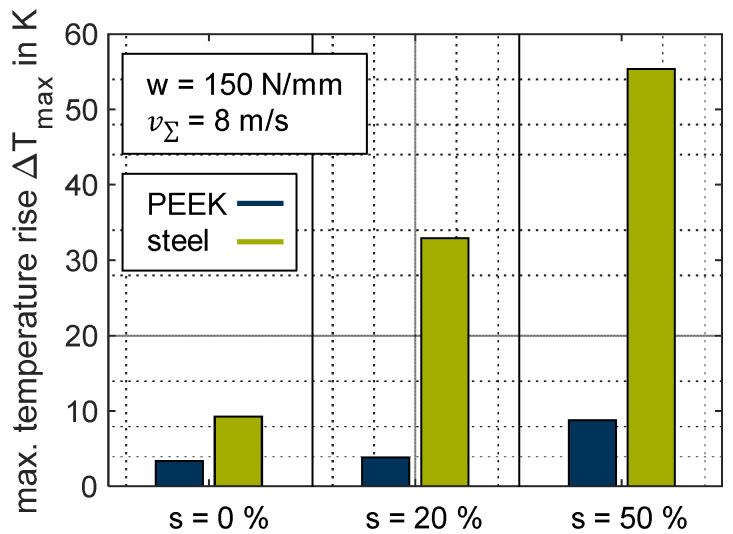
Maximum temperature rise ∆*T_max_* for different slip ratio *s* = {0; 20; 50}% for the thermoplastic EHL contact ZrO_2_/PEEK and steel EHL contact ZrO_2_/steel (line load *w* = 150 N/mm, sum velocity $v_{\sum }$ = 8 m/s, oil temperature *ϑ*_oil_ = 30 °C, MIN100).

Non-conformal contacts (such as those found in spur gears) are generally characterized by the high contact pressures resulting from the geometry and the material properties when using steel as a material. The maximum pressure in EHL contacts can reach several gigapascals in this case. The high Youngs’s modulus of steel or ceramic materials in particular favors high contact pressures. Thermoplastics used in gear applications typically have a Young’s modulus in the range of 800 to 3500 MPa and can therefore enable low contact pressures in non-conformal contacts in comparison to the line load. Figure 15 compares the pressure profile at a line load of *w* = 150 N/mm for the material pairings ZrO_2_/PEEK and ZrO_2_/steel at $v_{\sum }$ = 8 m/s and pure rolling (*s* = 0%). For better comparison, the contact pressure is shown in a dimensionless manner p/p_pmax_, and the sensor distance was normalized by the half Hertzian width *b_H_*. For the material pairing ZrO_2_/PEEK, *b_H_* was derived from the measurements, and for ZrO_2_/steel, it is calculated according to Hertzian theory [40] (values can be found in Table 1). Whereas the thermoplastic contact ZrO_2_/PEEK (blue) exhibits a typical hydrodynamic profile, the steel EHL contact ZrO_2_/steel contact shows a characteristic pressure build-up known for low-loaded steel contacts, which was measured by several authors using thin-film sensor technology, e.g., [32,39]. The line load of *w* = 150 N/mm results in a maximum contact pressure of *p_max_* ≈ 500 MPa and is therefore nearly a factor of six higher than the thermoplastic contact with 87 MPa. Comparable lubricated thermoplastics behavior, as contrasted with plain steel contacts, has been obtained by numerical modeling from Maier et al. [5]. In addition to the contact pressure, the temperature rise within the contact also significantly differs between the thermoplastic and steel EHL contact. In Figure 16, $\Delta T_{max}$ is shown for the line load *w* = 150 N/mm, sum velocity $v_{\sum }$ = 8 m/s and selected slip ratios of *s* = {0; 20; 50}%.

Under pure rolling (*s* = 0%), $\Delta T_{max}$ for the steel EHL Contact ZrO_2_/steel is already higher than at a slip ratio of *s* = 50% for the thermoplastic EHL contact ZrO_2_/PEEK. At its maximum, the steel EHL contact reaches 55 K, which is approximately a factor of six higher than the thermoplastic EHL contact at the operating point considered. This clearly shows that beyond thermal effusivity, the thermal behavior of EHL contacts is governed by the mechanical properties of the material. Furthermore, the thermal boundary conditions differ as the bulk temperature of the PEEK test disk with $\vartheta_{M,PEEK}$ ≈ 42 °C is higher compared to $\vartheta_{M,steel}$ ≈ 36 °C of the steel test disk, which can be attributed to the pronounced heat dissipation to the lubricant for the steel test disk.

## 5. Conclusions

In this study, the contact behavior of PEEK under elastohydrodynamic lubrication was analyzed. For this, in situ measurements of pressure and temperature distribution have been successfully performed using thin-film sensor technology. The main conclusions can be summarized as follows:

- The thermoplastic EHL contact with PEEK features lower maximum contact pressure and larger deformation of the contact zone than the reference steel EHL contact.
- The hydrodynamic pressure distribution with PEEK is not remarkably affected by speed and slip ratio. No indications of viscoelastic effects or loading frequency influence were observed.
- The temperature rise is very low despite the low thermal effusivity of PEEK. This can be attributed to the limited viscosity increase during contact, thus low fluid friction.
- The contact temperature distribution is determined by heat generation in the inlet zone and contact zone of the thermoplastic EHL contact with PEEK. The heat generation in the inlet zone governs the thermal behavior at low slip ratios, whereas the heat generation is governed within the contact zone at a high slip ratio.
- Thin-film sensor technology can be applied to thermoplastic EHL contacts. The ratio of absolute temperature to pressure rise is suitable with respect to the thermo- and piezoresistive measurement principle. Furthermore, the low mechanical stresses under fluid film lubrication shows no remarkable wear on the thin-film sensors.

Given that the thermoplastic PEEK exhibited relatively constant material properties over a wide range of temperatures, polyamide (PA) or polyoxymethylene (POM) can be subjects of investigation in further studies.

## Figures and Tables

**Table 1:** Mechanical and thermophysical properties of the considered test and sensor disks at room temperature (20 °C) [6,28,29,30].

	Poisson’s Ratio ν	Young’s Modulus *E* in MPa	Density ρ in kg/m^3^	Specific Heat Capacity *c_p_* in J/(kg K)	Thermal Conductivity λ in W/(m K)	Thermal Effusivity *e* in $J/(K\sqrt{s}m^{2})$
ZrO_2_ [29]	0.3	200,000	6000	400	2.50	2449
PEEK [6,30]	0.4	3400	1310	1100	0.29	624
Steel [28]	0.3	210,000	7760	431	44.00	12,131

**Table 2:** Temperature and pressure coefficients of the considered thin-film sensors.

Parameter	Platinum Sensor	Chromium Sensor
Temperature coefficient $\alpha_{T}$ in 1/k	$1.14\cdot 10^{-3}$	$0.17\cdot 10^{-3}$
Pressure coefficient $\alpha_{P}$ in mm^2^/N	$-1.10\cdot 10^{-5}$	$-4.50\cdot 10^{-5}$

**Table 3:** Considered operating conditions.

Parameter	
Line load w in N/mm	100, 150
Sum velocity $v_{\sum }$ in m/s	8, 12, 16
Slip ratio s in %	0, 5, 10, 20, 30, 40, 50
Oil injection temperature $\vartheta_{Oil}$ in °C	30

**Table 4:** Main properties of the oil considered.

ISO VG 100	
Kinematic viscosity ν at 40 °C in mm^2^/s	94.1
Kinematic viscosity ν at 40 °C in mm^2^/s	10.6
Viscosity index	95
Density ρ at 15 °C in kg/m^3^	884.5

## Data Availability

Not applicable.

## Abbreviations

EHL	Elastohydrodynamic lubrication
EP	Extreme-pressure
PEEK	Polyetheretherkethone
ZrO_2_	Ziconiumdioxide
PMMA	Poly (methyl methacrylate)
PA	Polyamide
PU	Polyurethane
POM	Polyoxymethylen

## Nomenclature

	**Unit**	**Description**
$\alpha_{p}$	mm^2^/N	Pressure coefficient
$\alpha_{T}$	1/K	Temperature coefficient
b_H_	mm	Half Hertzian contact width
D_I_	mm	Reduced diameter of curvature
E	N/mm^2^	Youngs’s modulus
E´	N/mm^2^	Reduced Young´s modulus
h_m_	nm	Minimum film thickness
s	%	Slip ratio
F_N_	N	Normal load
F_R_	N	Friction force
MS	1/s	Megasamples
p	N/mm^2^	Pressure
w	N/mm	Line load
T	K	Temperature
x	mm	Sensor distance
e	$J/\left(K\sqrt{s}m^{2}\right)$	Thermal effusivity
$l_{eff}$	mm	Effective length
$v_{\sum }$	m/s	Sum velocity
ϑ	°C	Temperature
$\vartheta_{M}$	°C	Bulk temperature
$\vartheta_{Oil}$	°C	Oil injection temperature
$\lambda_{rel}$	-	Lambda ratio
*µ*	-	Coefficient of friction
$\dot{v}$	L/min	Oil volume flow

## Indices

1	Upper/sensor disk
2	Lower/test disk
H	Hertzian
max	Maximum
S	Surface

## References

[1] de Vicente J., Stokes J., Spikes H. (2006). Soft lubrication of model hydrocolloids. Food Hydrocoll..

[2] Ziegltrum A., Maier E., Lohner T., Stahl K. (2020). A Numerical Study on Thermal Elastohydrodynamic Lubrication of Coated Polymers. Tribol. Lett..

[3] Myers T.G., Hall R.W., Savage M.D., Gaskell P.H. (1991). The transition region in elastohydrodynamic lubrication. Proc. R. A Math. Phys. Eng. Sci..

[4] Dearn K.D., Hoskins T., Andrei L., Walton D. (2013). Lubrication Regimes in High-Performance Polymer Spur Gears. Adv. Tribol..

[5] Maier E., Ziegltrum A., Lohner T., Stahl K. (2017). Characterization of TEHL contacts of thermoplastic gears. Forsch. Im Ing..

[6] Reitschuster S., Maier E., Lohner T., Stahl K. (2020). Friction and Temperature Behavior of Lubricated Thermoplastic Polymer Contacts. Lubricants.

[7] Dominghaus H. (2012). Kunststoffe: Eigenschaften und Anwendungen.

[8] Putignano C., Dini D. (2017). Soft Matter Lubrication: Does Solid Viscoelasticity Matter?. ACS Appl. Mater. Interfaces.

[9] Albahrani S., Philippon D., Vergne P., Bluet J. (2015). A review of in situ methodologies for studying elastohydrodynamic lubrication. Proc. Inst. Mech. Eng. Part J. J. Eng. Tribol..

[10] Turchina V., Sanborn D.M., Winer W.O. (1974). Temperature Measurements in Sliding Elastohydrodynamic lubrication. J. Lubr. Technol..

[11] Reddyhoff T., Spikes H.A., Olver A.V. (2009). Improved infrared temperature mapping of elastohydrodynamic contacts. J. Eng. Tribol..

[12] Lu J., Reddyhoff T., Dini D. (2017). 3D Measurements of Lubricant and Surface Temperatures Within an Elastohydrodynamic Contact. Tribol. Lett..

[13] Jubault I., Mansot J.L., Vergne P., Mazuyer D. (2001). In-situ Pressure Measurements Using Raman Microspectroscopy in a Rolling Elastohydrodynamic Contact. J. Tribol..

[14] Marx N., Guegan J., Spikes H. (2016). Elastohydrodynamic film thickness of soft EHL contacts using optical interferometry. Tribol. Int..

[15] Crook A.W. (1961). Elastohydrodynamic lubrication of roller. Nature.

[16] Kannel J.W., Bell J.C., Allen C.M. (1965). Methods for Determining Pressure Distributions in Lubricated Rolling Contact. ASLE Trans..

[17] Orcutt F.K. (1965). Experimental Study of Elastohydrodynamic Lubrication. ASLE Trans..

[18] Cheng H.S., Orcutt F.K. (1965). Paper 13: A Correlation between the Theoretical and Experimental Results on the Elastohydrodynamic Lubrication of Rolling and Sliding Contacts. Proc. Inst. Mech. Eng. Conf. Proc..

[19] Hamilton G.M., Moore S.L. (1971). Deformation and pressure in an elastohydrodynamic contact. Proc. R. Soc. Lond. Ser. A Math. Phys. Sci..

[20] Safa M., Anderson J., Leather J. (1983). Transducers for pressure, temperature and oil film thickness measurement in bearings. Sens. Actuators.

[21] Ebner M., Ziegltrum A., Lohner T., Michaelis K., Stahl K. (2018). Measurement of EHL temperature by thin film sensors—Thermal insulation effects. Tribol. Int..

[22] Dauber O. (2001). Elastohydrodynamische Rollreibung in Stahl-Keramik-Kontakten [Elastohydroydynamic Rolling Friction in Steel-Ceramic-Contacts]. Ph.D. Thesis.

[23] Safa M.M.A. (1982). Elastohydrodynamic Studies Using Thin Film Transducers. Ph.D. Thesis.

[24] Baumann H. (1985). Druck-und Temperaturmessungen mittels aufgedampfter Dünnschichtaufnehmer in einem elastohydrodynamischen Linienkontakt. Dissertation [Pressure- and Temperature Measurements with Evaporated Thin-Film Transducers in an Elastohydrodynamic Line Contact]. Ph.D. Thesis.

[25] Emmrich S., Plogmeyer M., Bartel D., Herrmann C. (2021). Development of a Thin-Film Sensor for In Situ Measurement of the Temperature Rise in Rolling Contacts with Fluid Film and Mixed Lubrication. Sensors.

[26] Hofmann S., Yilmaz M., Maier E., Lohner T., Stahl K. (2021). Friction and contact temperature in dry rolling-sliding contacts with MoS2-bonded and a-C:H:Zr DLC coatings. Int. J. Mech. Mater. Eng..

[27] Plogmeyer M., Gonzales G., Schulze V., Bräuer G. (2020). Development of thin-film based sensors for temperature and tool wear monitoring ruing machining. Tech. Mess..

[28] Deutsche Edelstahlwerke GmbH: Material Data Sheet of 1.7131/1.7139(16MnCr5/16MnCrS5). Witten. 2011. https://www.dew-stahl.com/fileadmin/files/dew-stahl.com/documents/Publikationen/Werkstoffdatenblaetter/Baustahl/1.7131_1.7139_de.pdf.

[29] Oxidkeramik J Cardenas GmbH: Produktdatenblatt [material data sheet], Albershausen, 2015. https://oxidkeramik.de/oxidkeramik-broschueren/Werkstoff-Datenblatt_OK997_CARSIC310_CR105_CR101.pdf.

[30] Campus- a Material Information System for the Plastics Industry, 2022. https://www.campusplastics.com/campus/de/datasheet/VESTAKEEP%C2%AE+L+4000+G/Evonik+Operations+GmbH/66/556ea1c9.

[31] Lohner T., Mayer J., Stahl K. EHL Contact Temperature—Comparison of Theoretical and Experimental Determination. Proceedings of the STLE 70th Annual Meeting & Exhibiiton.

[32] Simon M. (1984). Messung von elasto-hydrodynamischen Parametern und ihre Auswirkungen auf die Grübchentragfähigkeit vergüteter Scheiben und Zahnräder [Measurement of Elasto-Hydrodynamic Parameters and Their Impact on Pitting Carrying Capacity of Disks and Gears]. Ph.D. Thesis.

[33] Illenberger C.M., Tobie T., Stahl K. (2019). Flank-load-carrying-capacity of oil-lubricated thermoplastic gears for power transmission. Forsch. Im Ing..

[34] Laukotka E.M. (2007). FVA-Heft Nr. 660 Referenzöle Datensammlung.

[35] Niemann G., Winter H., Höhn B.-R., Stahl K. (2019). Maschinenelemente I—Konstruktion und Berechnung von Verbindungen, Lagern und Wellen.

[36] Dowson G., Higginson G.R. (1966). Elastohydrodynamic Lubrication, the Fundamentals of Roller and Gear Lubrication.

[37] Johnson K.L. (1970). Regimes of Elastohydrodynamic Lubrication. J. Mech. Eng. Sci..

[38] Marko H. (1980). Methoden der Systemtheorie.

[39] Kagerer E. (1991). Messung von elastohydrodynamischen Parametern im hochbelasteten Scheiben-und Zahnkontakt [Measurement of Elastohydrodynamic Parameters in Highly-Loaded Disk and Gear Contacts]. Ph.D. Thesis.

[40] Hertz H. (1882). Ueber die Berührung fester elastischer Körper. J. Für Die Reine Angew. Math..

